# Poly (vinyl alcohol)/hydroxyapatite nanocomposite films with efficient adsorption and oil-water separation capabilities

**DOI:** 10.3389/fchem.2026.1783228

**Published:** 2026-03-17

**Authors:** Sheng Liu, Mengyi Yuan, Ju Du, Yujuan Guo, Zushun Xu, Qing Li

**Affiliations:** 1 Ministry of Education Key Laboratory for the Green Preparation and Application of Functional Materials, Hubei Key Laboratory of Polymer Materials, School of Materials Science and Engineering, Hubei University, Wuhan, China; 2 Guangxi Key Laboratory of Advanced Structural Materials and Carbon Neutralization, Guangxi Colleges and Universities Key Laboratory of Environmental-Friendly Materials and New Technology for Carbon Neutralization, School of Materials and Environment, Guangxi Minzu University, Nanning, China

**Keywords:** adsorption catalysis, composites, hydroxyapatite, oil-water separation, Poly (vinyl alcohol)

## Abstract

This study used two adsorptive hydroxyapatite nanorods (HAPR) and hydroxyapatite nanowires (HAPW) as modifiers. HAPR and HAPW with different aspect ratios have different abilities to induce axial alignment of adjacent Poly (vinyl alcohol) (PVA) molecular chains as nucleation sites. Thus, they can regulate the comprehensive properties of the two composite films. Tensile curves show that the properties of the two composite films are optimal when the PVA/HAPR ratio is 25% and the PVA/HAPW ratio is 10%. Compared with pure PVA films, they are improved by 2.17 times and 1.62 times, respectively. In addition, the optimized PVA/HAPW composite film has an adsorption capacity of 26.2 g/g for 1-Pentanol. This is due to the microporous structure and surface groups constructed by HAPW. This study provides a new research idea for the preparation of PVA/HAPR and PVA/HAPW composite films with simple processes. It has potential scalability in the direction of achievable industrial oil-water separation materials.

## Graphical Abstract

**Figure:**
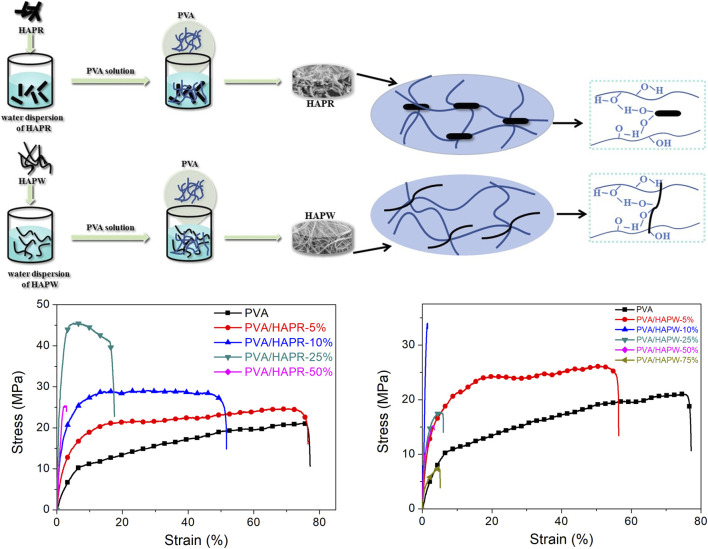

## 1 Introduction

With the increasing severity of environmental pollution, particularly water resource contamination, oil-water separation technology has attracted widespread attention in the field of water treatment (He and Guo, 2024; Jiao et al., 2025; Tao et al., 2023). These drawbacks restrict their widespread application in industrial production and environmental protection (Baig et al., 2025; Su et al., 2022; Wu et al., 2017). Traditional oil-water separation technologies face issues such as long residence time, high costs, and complex operations, and in practical applications, materials may suffer from pollution or clogging (Feng et al., 2024; Liu et al., 2022; Lu et al., 2024a; Lu et al., 2024b). These drawbacks limit their widespread use in industrial production and environmental protection. However, the membrane process can effectively solve these problems (Cheng et al., 2024; Ji et al., 2024; Zhang et al., 2025). Therefore, the development of low-cost, environmentally friendly, and high-performance oil-water separation materials is crucial. Poly (vinyl alcohol) (PVA) is a water-soluble polymer with a symmetric and orderly chain structure (Meera and Ramesan, 2025; Shabah et al., 2025). Due to the large number of hydroxyl groups in its molecular chain, stable hydrogen bonds are formed between molecules, resulting in a stable colloidal property, giving PVA excellent hydrophilicity, chemical stability, and film-forming ability (Ramesan et al., 2024; Ramesan et al., 2025). It is widely applied in coatings, adhesives, and food and pharmaceutical packaging (Fu et al., 2024; Xiang et al., 2024; Zhang et al., 2024). However, the adsorption and mechanical properties of pure PVA films are often not ideal, requiring the search for a material that can address these issues.

Currently, common strategies involve incorporating highly adsorbent materials into the PVA matrix, among which inorganic materials, carbon materials, and metal-organic frameworks have been widely studied (Ahmed et al., 2023; Joshi et al., 2024; Liang et al., 2025; Liu et al., 2023; Rethinasabapathy et al., 2025; Zhang et al., 2023; Zhuang and Wang, 2023). However, due to their poor stability and high cost, these materials are somewhat restricted in practical application (Zhao et al., 2025). Hydroxyapatite (HAP, Ca_10_(PO_4_)_6_(OH)_2_), an emerging and popular inorganic material used for bone substitution, has high stability and excellent adsorption properties (Biedrzycka and Skwarek, 2024; Fihri et al., 2017; Kong et al., 2020; Xia et al., 2019). However, pure HAP nanoparticles tend to agglomerate, along with other drawbacks (Ramesan et al., 2024; Ramesan and Surya, 2016). Since both HAP and PVA molecules contain hydroxyl groups, they can bond well via chemical interactions, improving the material’s adsorption and mechanical properties. Additionally, by altering the morphology and content of HAP in the PVA matrix, the composite material’s performance can be further modified.

Herein, two HAP nanomaterials with different morphologies, namely, hydroxyapatite nanorods (HAPR) and hydroxyapatite whiskers (HAPW), were used as modifiers and incorporated into the PVA matrix to fabricate a series of PVA/HAP nanocomposites. The incorporation of HAP introduces a certain number of pores, which reduces the density of the composite membrane while enhancing its adsorption capacity and flexibility. The morphology of the PVA/HAP nanocomposites was characterized by FESEM, and their adsorption performance for organic solvents and oil-water separation capability were measured. This study provides a novel solution for oil-water separation, which exhibits significant research value and broad application prospects.

## 2 Experimental section

### 2.1 Experimental reagent

Poly (vinyl alcohol) type 1799 (99%), sodium hydroxide (AR), methanol (AR), oleic acid (AR), anhydrous calcium chloride (AR), sodium dihydrogen phosphate (sodium dihydrogen phosphate) (AR), methanol (AR), n-amyl alcohol (AR), acetone (AR), trichloromethane (AR), oil red O (AR). All materials need no further processing prior to use.

### 2.2 Preparation of the hydroxyapatite nanorods

A 200 mL solution containing 8.713 g of disodium hydrogen phosphate dodecahydrate is added dropwise to 500 mL of a solution containing 4.500 g of calcium chloride while stirring at room temperature. The pH is adjusted and maintained at 11 with 1 M sodium hydroxide solution. The resulting mixed solution is then placed in a 70 °C water bath and stirred for 10 h. The product is collected by centrifugation, washed three times with deionized water, and the obtained HAPR are dispersed in deionized water for further use (Supplementary Figure S1).

### 2.3 Synthesis of the hydroxyapatite nanowires

A 150 mL solution containing 10.50 g of sodium hydroxide is mixed vigorously in an ice water bath with 60 mL of methanol, 105 mL of oleic acid, and 135 mL of deionized water. Then, 180 mL of a solution containing 9.36 g of monosodium phosphate and 120 mL of a solution containing 3.33 g of calcium chloride are added dropwise to the solution while stirring for 30 min. The resulting suspension is transferred to a reaction vessel and reacted at 180 °C for 24 h. The product is separated by centrifugation, washed several times with ethanol and deionized water, and then filtered through a 300-mesh sieve (Sun et al., 2017). The obtained HAPW are dispersed in deionized water for further use (Supplementary Figure S1).

### 2.4 Preparation of the PVA/HAP nanocomposites

Both the HAPR and HAPW dispersions tend to precipitate, so they need to be sonicated for 40 min before use. Then, according to the Tables 1, 2 the corresponding amounts of PVA, HAPR, and HAPW dispersions are weighed and mixed thoroughly. The mixture is stirred at 100 °C in an oil bath for 4.5 h to ensure complete dissolution. The resulting solutions are then poured into molds with circular frames. After the films are cast, they are quickly placed in a vacuum oven at 60 °C for 24 h and 80 °C for another 24 h to dry, and the molds are allowed to cool to room temperature before being peeled off. The resulting PVA/HAP nanocomposites with varying contents are named PVA, PVA/HAPR-5%, PVA/HAPR-10%, PVA/HAPR-25%, PVA/HAPR-50%, PVA/HAPW-5%, PVA/HAPW-10%, PVA/HAPW-25%, PVA/HAPW-50%, and PVA/HAPW-75%, according to the changes in the mass of HAPR and HAPW, with their contents expressed as weight percentages relative to the solid PVA matrix.

**TABLE 1:** Preparation and formulation of pure PVA and PVA/HAPR nanocomposites.

Sample	PVA (g)	HAPR dispersion solution (g)	Deionized water (mL)
PVA	0.80	0	40
PVA/HAPR-5%	0.76	2.23	38
PVA/HAPR-10%	0.72	4.47	36
PVA/HAPR-25%	0.60	11.16	29
PVA/HAPR-50%	0.40	22.33	18

**TABLE 2:** Preparation and formulation of pure PVA and PVA/HAPW nanocomposites.

Sample	PVA (g)	HAPW dispersion solution (g)	Deionized water (mL)
PVA	0.80	0	40
PVA/HAPW-5%	0.76	2.73	37
PVA/HAPW-10%	0.72	5.45	34
PVA/HAPW-25%	0.60	13.68	25
PVA/HAPW-50%	0.40	27.26	13
PVA/HAPW-75%	0.2	40.88	0

### 2.5 Adsorption experiments of PVA/HAP nanocomposites

To investigate the adsorption capacity of nanocomposites for organic solvents, the mass of different composite films was accurately weighed and designated as M_1_. The films were then immersed in 4 mL of organic solvents (toluene, acetone, chloroform, and 1-pentanol), and the adsorption system was sealed to prevent the evaporation of the solvents. After 2 h, the films were removed, the surface organic solvents were wiped off, and the mass was measured as M_2_. The adsorption rate (Q) was determined using the following Equation 1.

$$Q=\frac{M_{1}-M_{2}}{M_{1}}\times 100\%$$ (1)

### 2.6 Oil-water separation experiment

To verify the oil-water separation effect of high content composite films on toluene, nanocomposite films were selected as fillers to construct an organic wastewater filtration column. A plastic tube was used as a mold, leaving a portion of the wastewater area above and filling the bottom with composite films. A mixture of 2 mL of toluene (dyed red with oil red O) and 18 mL of water was used as the organic wastewater model. After stirring for 10 s, 2 mL of the mixture was added to the wastewater area for filtration. This process was repeated until all the solution had been filtered.

### 2.7 Characterization

The surface morphology of the catalysts were examined by field emission scanning electron microscope (FESEM), from Carl Zeiss Supra 55 at an accelerating voltage of 2 kV. Each sample was first diluted with a 60% ethanol aqueous solution at a mass ratio of 1 : 4000, followed by drying on standard silicon wafers. The mechanical properties were conducted by stress/strain test under uniaxial tension (5 mm min^-1^) using a JDL-10000N. The crystalline phases of samples were acquired by a Bruker D8 Advance Xray diffractometer (XRD, Cu-Kα radiation, λ = 1.541 Å).

## 3 Results and discussion

### 3.1 Analysis of HAP

**FIGURE 1:**
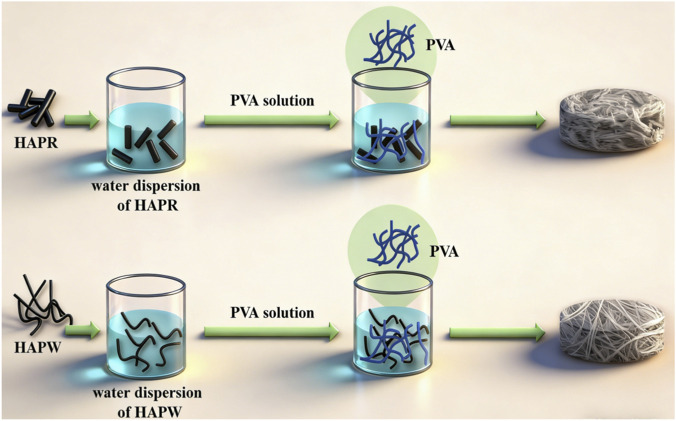
Scheme of the preparation of PVA/HAPR films and PVA/HAPW films.

**FIGURE 2:**
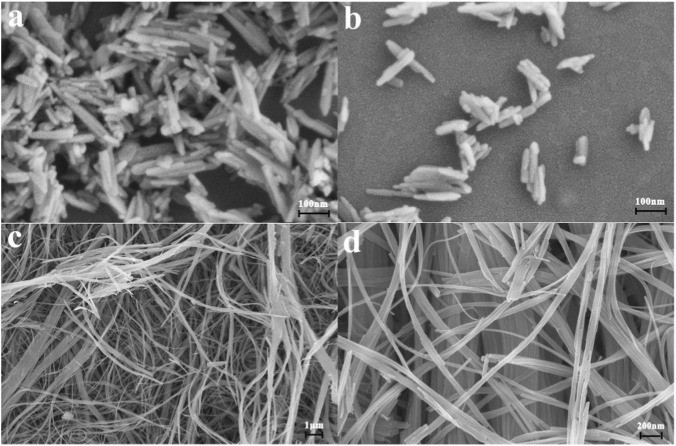
The FESEM images of **(a,b)** HAPR and **(c,d)** HAPW.

Figure 1 shows the preparation process of the two types of nanocomposite films. In Figures 2a-d, the hydroxyapatite nanoparticles under two different experimental conditions show different morphologies. Specifically, the HAPR nanoparticles are regular rod-shaped, with a length of about 100 nm, while the HAPW shows an overall fibrous structure with a length of several hundred micrometers and a diameter of approximately 25–100 nm. As shown in Supplementary Figures S2, S3, the characteristic peaks of HAP in the XRD spectra increase with the addition of both HAP types. Notably, the characteristic peaks of PVA are well preserved in the PVA/HAPW composite film, whereas they are hardly detectable in PVA/HAPR. This is because HAPR, with its elongated and granular morphology, blends more uniformly with PVA at lower concentrations. Consequently, it forms a continuous and ordered molecular chain network of PVA/HAPR, replacing the original PVA matrix structure.

### 3.2 The mechanical properties of PVA/HAP nanocomposites

**FIGURE 3:**
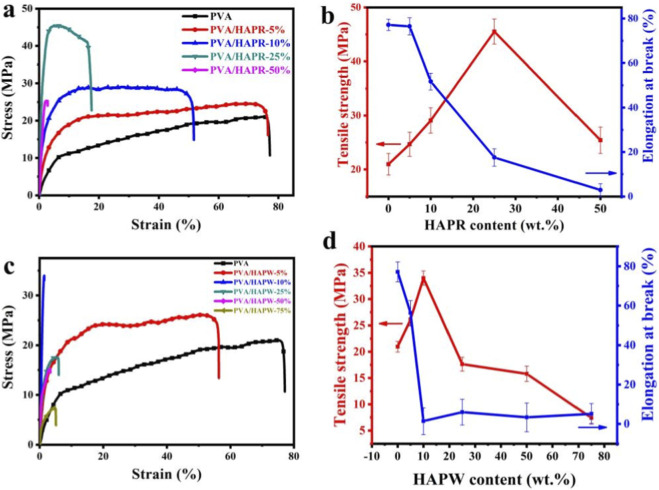
**(a,c)** Strain−stress curves of PVA/HAPR and PVA/HAPW nanocomposites; **(b,d)** Influences of PVA component on HAPR and HAPW nanocomposites’ elongation and tensile strength at rupture.

**FIGURE 4:**
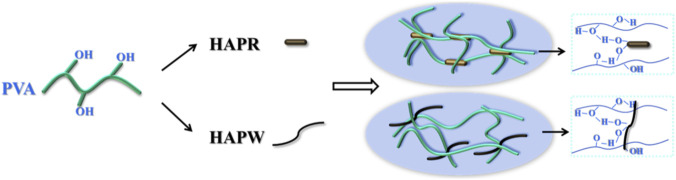
Schematic representation of the hydrogen bonds of the reaction between PVA/HAPR and PVA/HAPW.

To evaluate the mechanical properties of the composite films, the average tensile strength (σ) and elongation at break (ε_max_) were measured as evaluation standards. All samples were tested more than three times. As shown in Tables 3, 4, the tensile strength of PVA/HAPR and PVA/HAPW composite films both increased and then decreased with the content of HAPR and HAPW. When the content of HAPR reached 25%, the tensile strength of the composite film reached a maximum value of 45.54 MPa, showing the best mechanical performance. Similarly, when the HAPW content reached 10%, the tensile strength also reached the optimal value of 34 MPa. Surprisingly, all the composite films showed better mechanical properties than pure PVA. Furthermore, the elongation at break of PVA/HAP composite films decreased with the increasing HAP content, showing a negative correlation (Figure 3). This can mainly be attributed to the degree of bonding between the substrate material and the modifier. Due to the limited number of hydroxyl groups in the PVA substrate, only a small amount of HAP (HAPR≤25 wt%, HAPW≤10 wt%) forms bonds, while excessive HAP leads to aggregation due to the limited number of hydrogen bonds in the system, resulting in defects in PVA and a decline in the mechanical properties of the composite material. Additionally, the presence of hydrogen bonds limits the movement of the PVA molecular chains, causing the elongation at break to decrease (Li et al., 2020; Li et al., 2021; Li et al., 2023a; Li et al., 2023b; Szliszka et al., 2009). A schematic diagram of the hydrogen bonding between PVA and HAP is shown in Figure 4. Both HAPs exhibit excellent compatibility with PVA matrices via hydrogen bonding, acting as nucleation sites to induce the axial alignment of adjacent PVA molecular chains. Furthermore, owing to the shorter aspect ratio of HAPR compared with HAPW, HAPR blends more uniformly with PVA at lower concentrations, ultimately leading to the formation of a continuous and ordered PVA/HAPR molecular chain network (Shikha et al., 2022). This ordered network significantly increases the intermolecular entanglement density and anti-slip capacity, allowing the chain segments to bear stress collaboratively during stretching. As a result, improvements in mechanical properties can be achieved without the need for higher filler concentrations.

**TABLE 3:** The mechanical properties of pure PVA and PVA/HAPR nanocomposites.

Sample	Tensile strength (MPa)	Elongation at break (%)
PVA	20.97 ± 2.01	77.16 ± 2.52
PVA/HAPR-5%	24.68 ± 2.24	76.51 ± 3.84
PVA/HAPR-10%	29.1 ± 2.35	51.72 ± 3.73
PVA/HAPR-25%	45.54 ± 2.31	17.57 ± 3.93
PVA/HAPR-50%	25.43 ± 2.45	2.91 ± 2.82

**TABLE 4:** The mechanical properties of pure PVA and PVA/HAPW nanocomposites.

Sample	Tensile strength (MPa)	Elongation at break (%)
PVA	20.97 ± 2.01	77.16 ± 2.02
PVA/HAPW-5%	26.14 ± 1.24	56.37 ± 2.48
PVA/HAPW-10%	34.00 ± 1.35	1.49 ± 2.7
PVA/HAPW-25%	17.65 ± 1.31	6.04 ± 2.62
PVA/HAPW-50%	15.81 ± 1.45	3.41 ± 2.9
PVA/HAPW-75%	7.41 ± 1.04	3.41 ± 2.08

### 3.3 The adsorption properties of PVA/HAP nanocomposites

**FIGURE 5:**
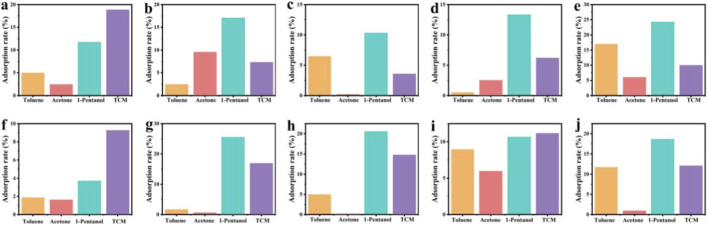
The adsorption rate of PVA/HAP nanocomposites to organic solvents (Toluene, Acetone, 1-Pentanol, Trichloromethane (TCM)): **(a)** pure PVA; **(b)** PVA/HAPR-5%; **(c)** PVA/HAPR-10%; **(d)** PVA/HAPR-25%; **(e)** PVA/HAPR-50%; **(f)** PVA/HAPW-5%; **(g)** PVA/HAPW-10%; **(h)** PVA/HAPW-25%; **(i)** PVA/HAPW-50%; **(j)** PVA/HAPW-75%.

The measurements showed (Figure 5) that both PVA and composite films have certain adsorption capabilities for organic solvents (toluene, acetone, 1-pentanol, and chloroform), and the adsorption rate of the composite films for toluene varied with the increasing content of HAPR and HAPW, reaching maximum values of 16.99% and 11.70% when HAPR content was 50% and HAPW content was 75%, respectively. For acetone, the adsorption performance of both composite films showed a downward trend, while the adsorption rate for 1-pentanol was relatively good. Among them, the PVA/HAPR-50% composite film reached a maximum adsorption rate of 24.25%, and the PVA/HAPW-10% reached 25.53%. Lastly, for chloroform, a downward trend in adsorption was observed, but both exhibited feasible adsorption capabilities. In summary, the composite films with different ratios showed certain adsorption rates for toluene, acetone, 1-pentanol, and chloroform, with lower adsorption rates for acetone and better adsorption rates for toluene, 1-pentanol, and chloroform. This is mainly attributed to the addition of HAPR and HAPW, which endowed the composite material with good adsorption performance (He et al., 2016). The introduction of HAPR and HAPW particles can disrupt the tight packing of PVA molecular chains, generate more interconnected microporous and mesoporous structures within the composite membrane, and markedly increase its specific surface area and pore volume. This modification provides additional physical adsorption sites for organic molecules, while the morphological differences between HAPR and HAPW directly influence the adsorption mechanism of the composite membrane. Furthermore, the hydroxyl and phosphate groups on the surface of hydrophobic HAP can enhance the adsorption of polar organic compounds (acetone and 1-pentanol) via electrostatic interactions. In contrast, the adsorption of chloroform primarily depends on physical adsorption, hydrophobic interactions, and weak ion exchange. When an appropriate amount of these two HAP types is added, these three mechanisms act synergistically to enhance adsorption. Conversely, excessive HAP addition results in pore blockage and shielding of ion exchange sites, thereby reducing adsorption efficiency.

### 3.4 Oil-water separation performance of PVA/HAP nanocomposites

**FIGURE 6:**
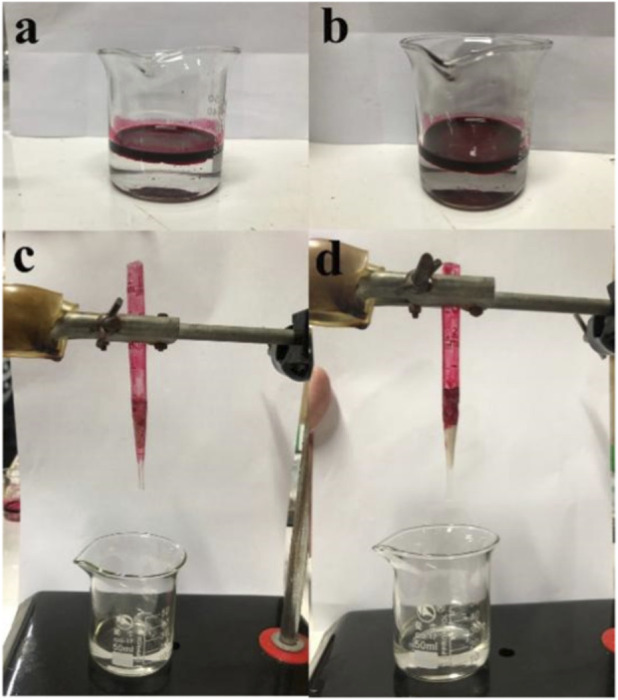
**(a,b)** Organic contaminated water stained with toluene (oil red O); **(c,d)** PVA/HAPR and PVA/HAPW nanocomposites oil-water separation effect diagram.

As shown in Figure 6, when the filtration column filled with composite films was used to filter a mixture of toluene (dyed with oil red O) and water, toluene was absorbed by the PVA/HAP composite films column and could not pass through, while water was completely separated from toluene. This is mainly because HAPR and HAPW themselves have good adsorption properties, and their incorporation into PVA provided the composite film with excellent adsorption performance for oil-water separation. The film form also facilitated subsequent recovery and reuse, enhancing the green recycling advantage of the experiment. Furthermore, as shown in Supplementary Table S1 and Supplementary Figure S4, the prepared composite membrane exhibits superior performance compared to those reported in the literature, demonstrating excellent cycling stability.

## 4 Conclusion

Since the two nanomaterials exhibit distinct aspect ratios, they were subsequently incorporated into PVA films. When bonded to the PVA matrix through hydrogen bonding, they can serve as nucleation sites. These nucleation sites induce distinct axial alignments of adjacent PVA molecular chains. An ordered network structure is thereby formed. This significantly enhances the intermolecular entanglement density and anti-slip capacity. Consequently, the mechanical properties of the composite films are enhanced. In the PVA/HAPR-25% and PVA/HAPW-10% systems, their mechanical properties are 2.17 and 1.62 times those of pure PVA films, respectively. Owing to the adsorptive properties of nanomaterials, the composites exhibit excellent adsorption capacities for various organic solvents. Additionally, they enable effective oil-water separation between toluene and water. The film morphology further enhances the recyclability of the composites. These excellent comprehensive properties endow PVA/HAPR and PVA/HAPW composite films with great potential as industrially applicable, high-efficiency oil-water separation materials. They also offer insights into the high-value utilization of HAP with different morphologies.

## Data Availability

The original contributions presented in the study are included in the article/Supplementary Material, further inquiries can be directed to the corresponding authors.
