# Supplementary material for: Poly (vinyl alcohol)/hydroxyapatite nanocomposite films with efficient adsorption and oil-water separation capabilities
Source: Front Chem. 2026 Mar 17;14:1783228. doi: 10.3389/fchem.2026.1783228 (PMC13036187; doi:10.3389/fchem.2026.1783228)
Supplement: Supplementary file 1 [file DataSheet1.docx]

## Supporting Information

## Poly (vinyl alcohol)/hydroxyapatite nanocomposite films with efficient adsorption and oil-water separation capabilities

Sheng Liu^a,1^, Mengyi Yuan^a,1^, Ju Du^b,*^, Yujuan Guo^a^, Zushun Xu^a^, Qing Li^a,*^

^a^ Ministry of Education Key Laboratory for the Green Preparation and Application of Functional Materials, Hubei Key Laboratory of Polymer Materials, School of Materials Science and Engineering, Hubei University, Wuhan 430062, China.

^b^ Guangxi Key Laboratory of Advanced Structural Materials and Carbon Neutralization, Guangxi Colleges and Universities Key Laboratory of Environmental-friendly Materials and New Technology for Carbon Neutralization, School of Materials and Environment, Guangxi Minzu University, Nanning 530105，China.

*Corresponding authors. E-mail addresses: [20219168@gxmzu.edu.cn](mailto:20219168@gxmzu.edu.cn) (J. Du), [liqing@hubu.edu.cn](mailto:liqing@hubu.edu.cn) (Q. Li)

^1^ These three authors contributed equally to this work.


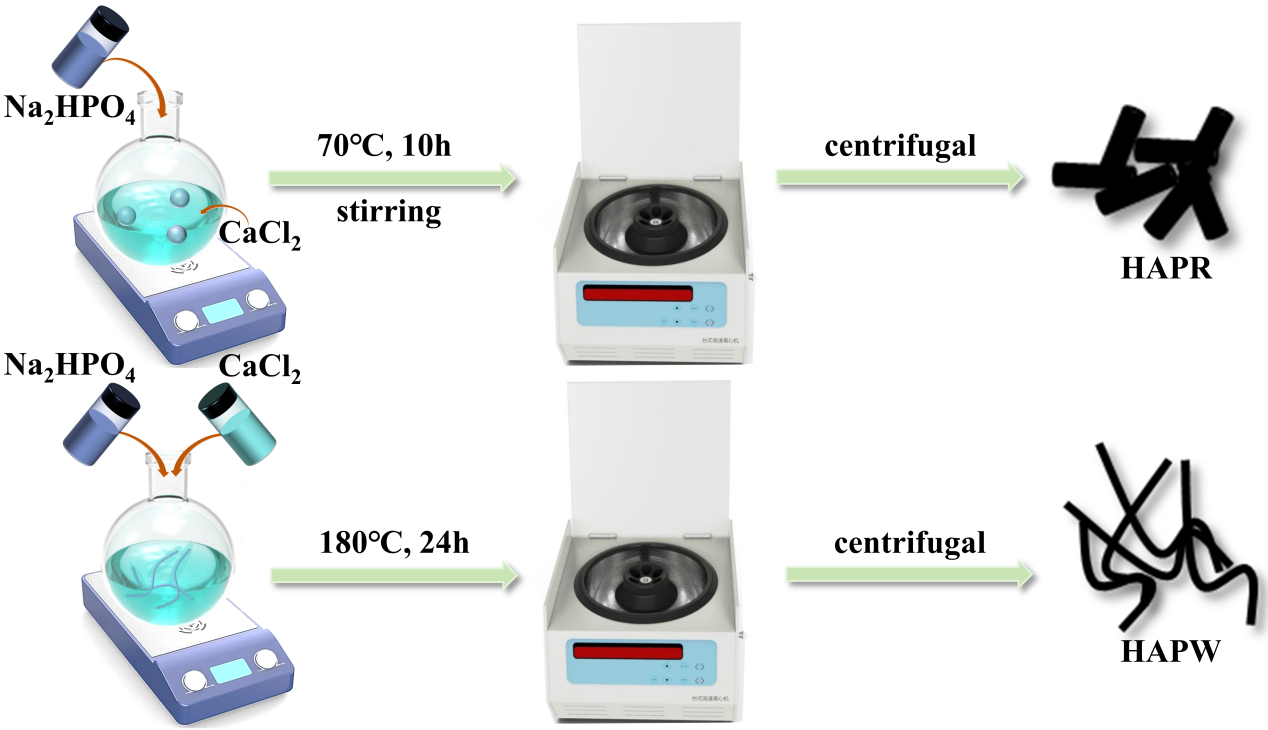


**Figure S1.** Scheme of the preparation of HAPR films and HAPW.


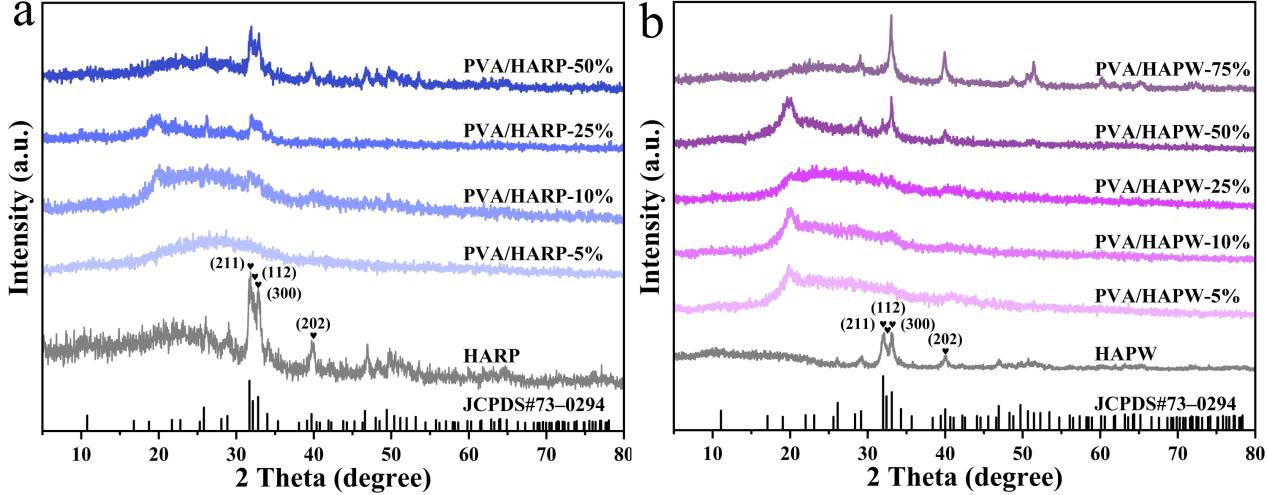


**Figure S2.** X-ray diffractometer (XRD) patterns of the as-prepared samples.


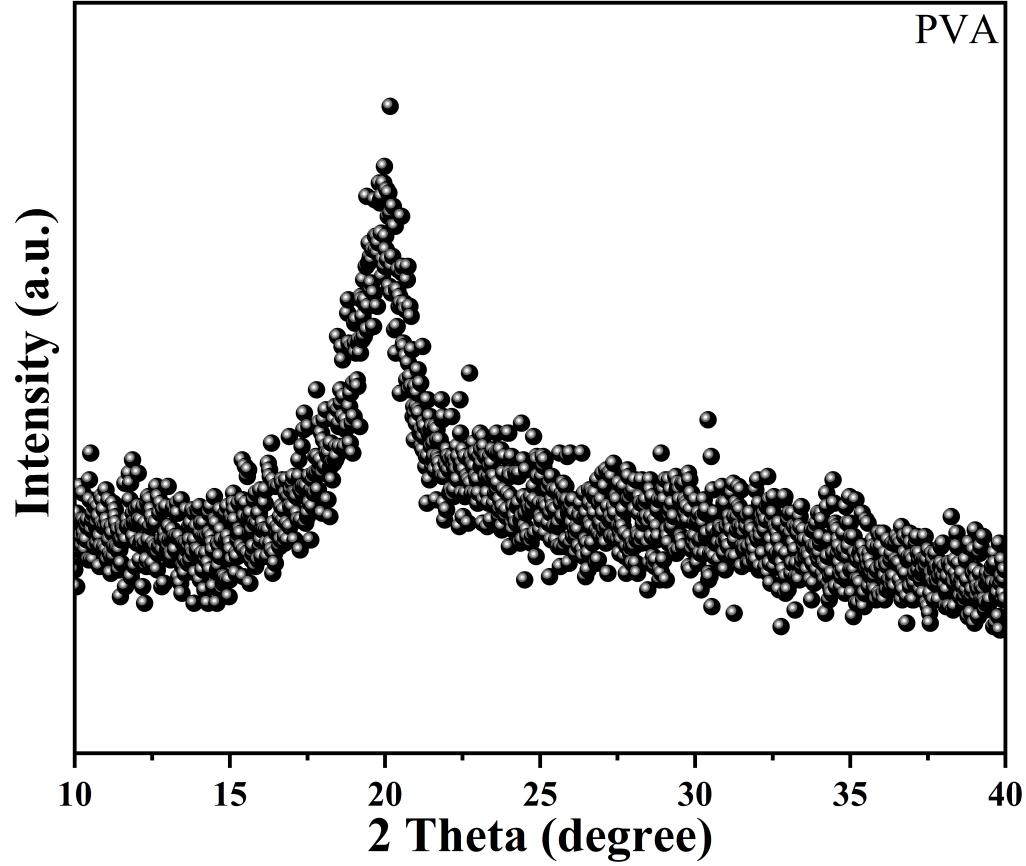


**Figure S3.** XRD patterns of PVA.

**Table S1.** Partial reports on materials used for the absorption of chloroform and toluene are not detailed in this study.

| Samples | Year | Chloroform (g/g) | Toluene (g/g) | Ref. |
| --- | --- | --- | --- | --- |
| nano-HAP/polyurethane | 2021 | 22.7 | – | (Liu et al., 2021) |
| hydrophobic thiolated graphene | 2017 | 52.5 | 51.2 | (Zhang et al., 2017) |
| sodium carboxymethyl cellulose/2,2,6,6-tetramethylpiperidinyl-1-oxyl/mediated oxidized cellulose | 2025 | 21.2 | – | (Yang et al., 2025) |
| sodium alginate/sodium carboxymethyl cellulose | 2023 | 8.2– | – | (Li et al., 2023) |
| porous reduced graphene oxide/poly(ethyleneimine)/polydimethylsiloxane | 2022 | 7.4 | 16.2 | (Tong et al., 2022) |
| PVA/HAP | 2026 | 19.8 | 17.0 | this work |


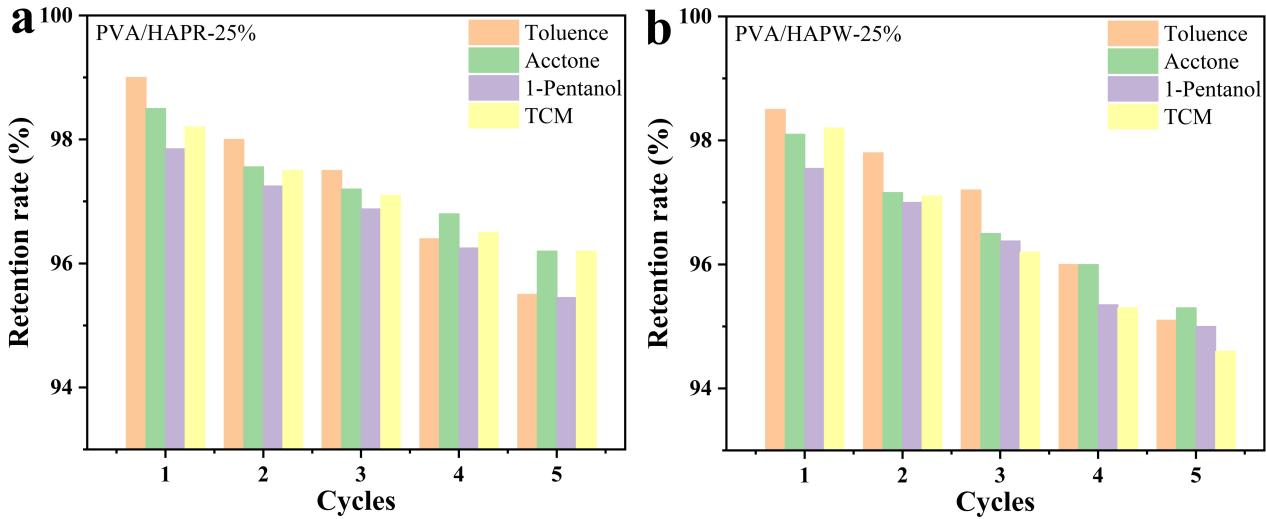


**Figure S4.** 5 cycles of adsorption experiments (a) PVA/HAPR-25%, (b) PVA/HAPW-25%.

**Reference**

Li, H., Huang, J., Shen, S., Meng, C., Wang, H. and Fu, J. (2023). Superhydrophobic sodium alginate/cellulose aerogel for dye adsorption and oil–water separation. *Cellulose* 30(11), 7157-7175. doi.org/10.1007/s10570-023-05307-4.

Liu, C., Chen, S.-H., Yang-Zhou, C.-H., Zhang, Q.-G. and Michael, R.N. (2021). Application of nano-hydroxyapatite derived from oyster shell in fabricating superhydrophobic sponge for efficient oil/water separation. *Molecules* 26(12), 3703. doi.org/10.3390/molecules26123703.

Tong, H., Chen, H., Zhao, Y., Liu, M., Cheng, Y., Lu, J., Tao, Y., Du, J. and Wang, H. (2022). Robust PDMS-based porous sponge with enhanced recyclability for selective separation of oil-water mixture. *Colloid. Surfaces A: Phy. Eng. Aspect*. 648, 129228. doi.org/10.1016/j.colsurfa.2022.129228.

Yang, R., Zhen, Y., Qin, J., Gao, H., Fong, I.L., Khong, H.Y., El-Sesy, M.E. and Zhao, Y. (2025). Preparation of nanocellulose-based aerogel by bidirectional freezing method for high performance oil-water separation. *J. Environ. Chem. Eng*. 13, 118967. doi.org/10.1016/j.jece.2025.118967.

Zhang, L., Li, H., Lai, X., Su, X., Liang, T. and Zeng, X. (2017). Thiolated graphene-based superhydrophobic sponges for oil-water separation. *Che. Eng. J.* 316, 736-743. dx.doi.org/10.1016/j.cej.2017.02.030.
